# MoHRiPA—An Architecture for Hybrid Resources Management of Private Cloud Environments

**DOI:** 10.3390/s21206857

**Published:** 2021-10-15

**Authors:** Gabriel Tomiatti Andreazi, Júlio Cezar Estrella, Sarita Mazzini Bruschi, Roger Immich, Daniel Guidoni, Lourenço Alves Pereira Júnior, Rodolfo Ipolito Meneguette

**Affiliations:** 1 Computer System Division, Institute of Mathematics and Computer Science, University of São Paulo, São Paulo 13560-970, SP, Brazil; tomiatti@usp.br (G.T.A.); jcezar@icmc.usp.br (J.C.E.); sarita@icmc.usp.br (S.M.B.); meneguette@icmc.usp.br (R.I.M.); 2 Computer Science Division, Federal University of Rio Grande do Norte, Natal 59078-970, RN, Brazil; roger@imd.ufrn.br; 3 Computer Science Division, Federal University of São João del-Rei, São João del-Rei 36301-360, MG, Brazil; guidoni@ufsj.edu.br; 4 Computer Science Division, Aeronautics Institute of Technology (ITA), São José dos Campos 12228-900, SP, Brazil

**Keywords:** cloud computing, service provider, resource management

## Abstract

The high demand for data processing in web applications has grown in recent years due to the increased computing infrastructure supply as a service in a cloud computing ecosystem. This ecosystem offers benefits such as broad network access, elasticity, and resource sharing, among others. However, properly exploiting these benefits requires optimized provisioning of computational resources in the target infrastructure. Several studies in the literature improve the quality of this management, which involves enhancing the scalability of the infrastructure, either through cost management policies or strategies aimed at resource scaling. However, few studies adequately explore performance evaluation mechanisms. In this context, we present the MoHRiPA—Management of Hybrid Resources in Private cloud Architecture. MoHRiPA has a modular design encompassing scheduling algorithms, virtualization tools, and monitoring tools. The proposed architecture solution allows assessing the overall system’s performance by using complete factorial planning to identify the general behavior of architecture under high demand of requests. It also evaluates workload behavior, the number of virtualized resources, and provides an elastic resource manager. A composite metric is also proposed and adopted as a criterion for resource scaling. This work presents a performance evaluation by using formal techniques, which analyses the scheduling algorithms of architecture and the experiment bottlenecks analysis, average response time, and latency. In summary, the proposed MoHRiPA mapping resources algorithm (HashRefresh) showed significant improvement results than the analyzed competitor, decreasing about 7% percent in the uniform average compared to ListSheduling (LS).

## 1. Introduction

The quantity and quality of information are crucial for the decision-making process. However, some problems require high processing time, resulting in excessive spending in terms of cost. Therefore, proper data processing and information generation must consider a High Performance and Distributed Computing environment. To this end, it is necessary to consider the availability of computational resources, the workload imposed on the applications, and resource scaling mechanisms in conjunction with the computational infrastructure model of the adopted environment.

Infrastructure as a service (IaaS) has been one of the fastest-growing areas in the past [1], enabling on-demand infrastructure provisioning. For an administrator, this opens up an opportunity to store some service or data set, as it considers the cost of only contracting the service without the need to worry about energy [2], equipment maintenance, refrigeration, and connectivity, among other resources [3].

Cloud computing centers entail high investment for both companies and users. Therefore, it is necessary to have responsible infrastructure management; after all, the customer must receive satisfactory service and look for more efficient, conscious, and economical methods of using the resources [4,5,6,7,8,9]. The customer is making a large investment in this type of service and will always seek the best cost relative to benefits received. The service provider will also seek to perform a service in the best possible way in order to serve with the highest quality [7,10,11,12,13]. Any business must serve better, especially when there is a contract on the provider’s part to guarantee services, allocate resources, map resources, and adapt resources. Therefore, the cloud infrastructure must have efficient resource management in order to not break contracts and lose customers.

This work proposes the Management of Hybrid Resources in Private Cloud Architecture (MoHRiPA) that provides services through distributed modules designed for high-performance allocation using different scheduling techniques. MoHRiPA has resource management modules such as Resource Provider (RP) and the Manager of Elastic Resource (MER) responsible for acquiring and allocating resources in the cloud. Furthermore, the architecture features a component that performs the monitoring of the Log Server (LS) system and monitors the use of available resources with Resource Monitor (RM). Unlike other solutions, MoHRiPA uses the metric composed of performance index, which equates to different data such as CPU, memory, disk, and network data (because of that, there are more opportunities to analyze heterogeneous metrics and to provide a richer mapping consistent with resources’ current performance). Thus, MoHRiPA can act with the provision of services and allocation of virtual machines. By running a comparison with architecture using ListScheduling to MoHRiPA using HashRefresh, the proposed solution performed better because HashRefresh updates the resource pool by updates and hashes. Its update is performed by request and not by time scheduling, such as the ListScheduling.

Therefore, the major contributions of this investigation are as follows:

- Propose an architecture of cloud resources that operate in IaaS and PaaS environments;
- Propose a mapping algorithm that considers the total performance of the physical machine to improve the overall system execution;
- The study and application of different algorithms of resource mapping as ListScheduling and HashRefresh for resource allocation and management;
- Discuss the approaches in the literature concerning our solution, considering differences and similarities in monitoring processes and policies;
- Survey requirements for implementing heterogeneous ranking algorithms for other platforms.

This work is structured as follows. Section 2 present the related work. The proposed architecture and principal solution concepts are discussed in Section 3. Section 4 provides performance evaluation. Finally, Section 6 discusses the conclusion of the paper and future investigations.

## 2. Related Work

There are several works in the literature about scalability [5,14,15,16,17,18,19,20,21,22,23,24,25]. However, they tend to focus on mechanisms that contribute to infrastructure robustness [14] since carrying out economic management of resources directly impacts the service’s quality. Management policies of consumption encourage clients to use the infrastructure, such as the architectural model presented.

The authors of [15] propose a method of scheduling by prioritizing tasks in parallel. This approach uses the concept of master/slave as a technique. The proposed algorithm improves performance and resource utilization, in addition to reducing execution time during load balancing. There is the master who receives all tasks through the task manager and distributes them to the slave machines that perform these jobs on their virtual machines. However, the scheduling of tasks by the master node can utilize two scheduling algorithms, Round-Robin and First In, First Out (FIFO), when considering only the delivery of tasks and not the states of the slave nodes. Such an approach can overload the computational resources available. After all, in a real-world environment, only considering task distribution is not ideal, and it is essential to consider the workload imposed on slave nodes.

In [16], the authors propose a framework for the allocation of automated resources via task scheduler based on deep learning. The scheduler worked according to SLA’s feedback, prioritizing the requests and increasing the response performance. However, non-adaptive SLAs bound the architecture capacity as faulty specification potentially results in inaccurate allocations. Therefore, to work without this obstacle, the solution should have an infrastructure reconfiguration module. If the configurations are not available, there is a reconfiguration of the components, thus scheduling the tasks again. The neural network is dependent on feedback so that an auxiliary solution can solve this problem.

The authors [17] use different algorithms for routing and non-preemptive scheduling jobs with variable and unknown sizes in a cloud-computing data center. The focus is the usability of these algorithms, choosing a MaxWeight schedule in either local or global refresh times. Nevertheless, the paper demonstrates the effectiveness of different refresh times by equations and methods. However, the method focused on routing and scheduling algorithms, while our proposal contemplates resource management.

In [19], the authors proposed a heuristic approach that combines a modified analytical hierarchy process analysis (MAHP), bandwidth-aware divisible programming (BATS) + BAR optimization, divide-and-conquer methods to perform a task, and resource scheduling. These different approaches process each task before allocating resources in the cloud using the combined optimization BATS + BAR. Another approach is a divide-and-conquer approach that compares existing BATS by the evolution algorithm differential (IDEA) to determine the turnaround and response times. However, this approach relies on task prediction allocation. Therefore, when a task is missing available VMs until the task is assigned to run, the process will be waiting for a response; in worst cases, this could take time.

The work in [20] seeks to optimize task scheduling and resource allocation by using a combination of Taguchim’s method and the differential evolutionary algorithm (DEA). Thus, the referred method optimizes the allocation of tasks by the resource provider. However, the strategy of the article was aimed at optimizing the tasks and neither included an economic policy of resources nor did it present a method of reducing idleness based on resources. The approach in our proposal addresses the concept of the policy of saving virtual resources by utilizing heterogeneous information ranking methods for the allocation of services.

The authors of [21] establish the reduction in keeping physical machines connected in their work, as providers leave physical machines connected and allocate Virtual Machines (VMs) according to demand. The demand is elastic, as it may occur at certain times of the day, and the demand decreases, leaving idle resources. This work uses the Markov chain to optimize the use of the infrastructure by determining, through the use of CPU, which machines could perform a migration of VM’s to decrease the number of physical machines connected simultaneously. The environment used was CloudSim, and the concept behind the proposal presents some critical issues. Using CPU as a decision metric may not be the only metric needed to determine which machines consume more or fewer resources. There are different types of applications, and some consume more RAM than a CPU. However, when considering a composite metric as the performance index proposed by [26], this would have a more volatile metric for the optimization of any scheduling considering performance. Another point is about shutting down physical machines in the simulation environment. They were considered feasible in real solutions such as the datacenter cloud, as they require an operation of 24/7 availability. Only particular events (programmed maintenance or component replacement) resulted in machine turn off. Specific solutions use virtual images downloaded to their volatile disk. It incurs an overhead during the boot time due to the network overhead and operating system image propagation. Consequently, it requires a mechanism to avoid the on and off behavior.

The work [23] proposes a hierarchical framework for allocating virtual resources and cloud energy management through a decision-making system with Deep Reinforcement Learning (DRL). This work has a policy that aims to minimize energy consumption while maintaining performance viable within the infrastructure. The learning model consists of mapping all available servers and over the current jobs, helping the determination of which job to submit to a server with a lower load, thus not generating overhead to the server. Nevertheless, reinforcement via deep learning happens with each interaction that maintains a greater consistency in the quality and speed of the mappings. Using the neural network as an index, what will be performed is established based on the previous interactions. However, this method did not address an architectural model, and it only addressed the resource provider’s profound learning actuator module.

The challenge addressed by [24] is to carry out a trade-off between load balancing and Quality of Service (QoS). The priority was to balance load without compromising these operations, provided resource allocation and migration policies. One segment deals with the ordering of tasks based on the size of the task and the limit that the infrastructure can provide without losing performance. Therefore, the other segment applies the change in the use of physical hosts in demand to migrate workloads to other machines; thus, a load of an overloaded physical host migrates virtual machines to another provider, decreasing the workload consequently. Both methodologies show promising results. Nevertheless, the simulation approach precludes the insertion of realistic scenarios, such as the time needed to update the mapping of resources and ordering and updating them with a list scheduling algorithm as needed.

In [5], a model was proposed to encourage the use of resources at a reduced cost due to processing demand, with a bonus made from the moment the infrastructure can increase the workload as there is no shortage of resources. The model is attractive because it generates an advantage in reducing customer costs and deals with the provider’s idleness due to the incentive to use the architecture. However, the model does not portray the architectural model, generating particular concerns about its functioning, such as the following: How would the control of resource scarcity be carried out? Furthermore, since monitoring is essential in this model, it is rarely mentioned, and issues about the granularity of monitoring information, which theoretically can impact the model, are not adequately described. Thus, the control of scarcity of resources demands information that corroborates with the deepening of this method.

In [25], the authors perform the expansion of resource allocation according to demand via automated exchanges between providers. The main factor is the collaboration between the providers, which forces transactions on idle resources to occur among themselves. Agents select the provider with the lower workload to meet the new demand, allowing proper load balancing between providers. The decision-making process relies on scoring metrics, and the provider is chosen based on the response time, performance, elasticity, availability, and compliance with the Service Level Agreement (SLA). However, the model still presents a charging system based on the provider transaction and weighs the urgency, transaction time, and resource consolidation variables. The model showed satisfactory results during task scheduling and measuring a reduced fee concerning what the providers charge for the transaction of resources. If the providers have no candidate providers to be elected, the architecture concept would be paralyzed or escalate unreliable tasks, interfering in the SLA. Consequently, if there is a prediction of when the providers will have completed jobs, it could be pre-allocated to a queue; thus, there would be no architecture downtime.

Concerning the management of resources, the proposed work adopts the provision of services (SaaS) or the provision of virtual machines (IaaS). The works found in the literature studied this concept differently as resource management begins in requisition, and fluctuations in demand shape the computing infrastructure needs with respect to monitoring metrics, such as CPU or power consumption. Some jobs make decisions only based on applications executing CPU-bound operations, making specific solutions ineffective considering that many applications have high memory consumption or high memory and CPU consumption. Composed metrics such as those used in the proposal of [27] add more information such as network and disk; thus, they tend to provide richer management information, enabling the management of infrastructure resources more professionally.

Table 1 shows a compilation of the discussed resource management approaches, showing the economic policies and the category of resource manager and monitor adopted.

Compared with literature solutions, the proposed architecture concerning economic policies has the principle of generating a reduction in idleness and resource consumption. However, idle reduction consists of several techniques, such as assigning tasks in a distributed manner, thus avoiding host overload. This control is essential to determine what and where it is being executed. Another example includes loads: if an application starts loading data, its load will increase with the processing of that data. Thus, the granularity of information on jobs performed and information collected by the resource monitor is required in order to deal with idleness. Thus, one of the main points to be analyzed to establish an economic policy is the consumption of heterogeneous resources. Resource scheduling is one of the crucial factors when assigning tasks to the infrastructure. It requires proper task scheduling; otherwise, the solution cannot respond to all tasks or result in an overloaded system. In the worst cases, violating service contracts with the user may occur, decreasing the processing capacity and generating bottlenecks in the network. Numerous techniques use algorithms that provide the best information to allocate in the best possible way. In some cases, migration is the post-allocation step, and it is necessary to migrate processing from one place to another. The suggested proposal is to allocate resources that observe the entire infrastructure so that the infrastructure scales according to demand.

## 3. Proposed Architecture

**Figure 1:**
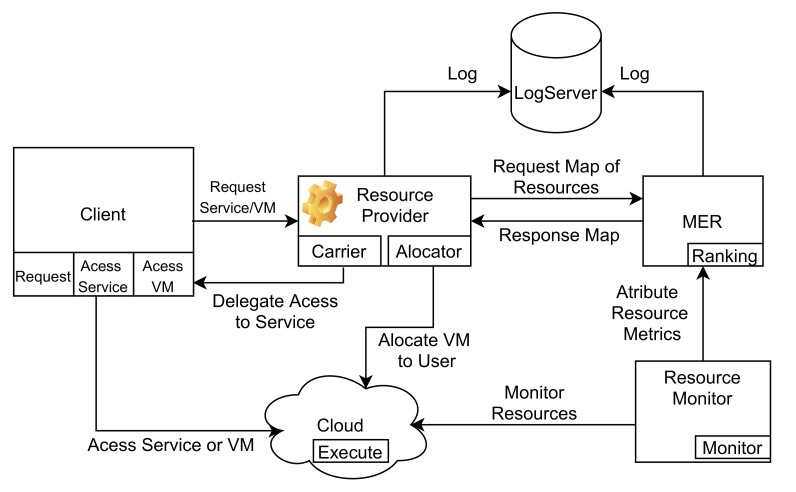
MoHRiPA architecture.

This section describes MoHRiPA—An Architecture for Hybrid Resources Management of Private Cloud Environments—which follows service-oriented architecture [26] and focuses on attending requests from clients by using a broker selector for services while considering the dynamic provisioning of computational resources in the service providers. On the other hand, the MoHRiPA model can control which services are offered efficiently. MoHRiPA has four modules, as demonstrated in Figure 1. First, the client has the responsibility for requesting and accessing services or VM. A request results in a resource provider (RP), which acts as a broker. The RP is responsible for service orchestration and management of configurations such as carrier service and allocation. Finally, the manager of elastic resources (MER) is responsible for auditing the performance metrics. These metrics are monitored by the Resource Monitor(RM), and the aggregation of these metrics consists in mapping the pools of resources consumed by RP; mapping algorithms calculate these pools.

**Figure 2:**
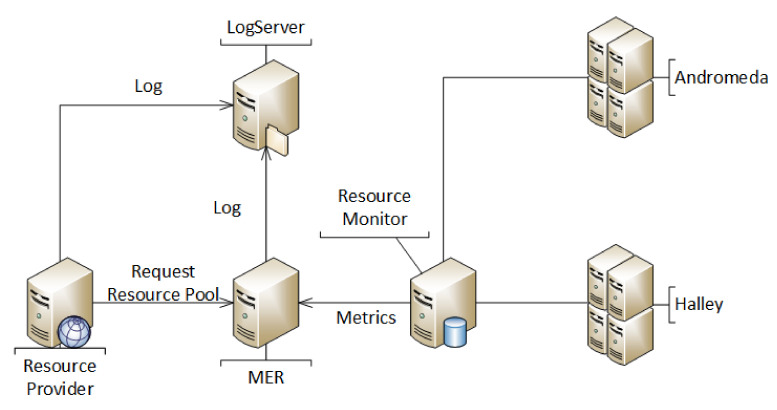
Conceptual model of MoHRiPA.

MoHRiPA consists of a distributed components architecture; thus, the servers of a company can attend to simultaneous clients. Figure 2 demonstrates the topology of components of MoHRiPA. Thus, the scheduling algorithms are on the Manager of Elastic Resources (MER) module. Virtualization tools were installed on each infrastructure machine in the architecture and manipulated by the Resource Provider (RP) module that is responsible for commands such as switching on, switching off, pausing, and returning.

**Figure 3:**
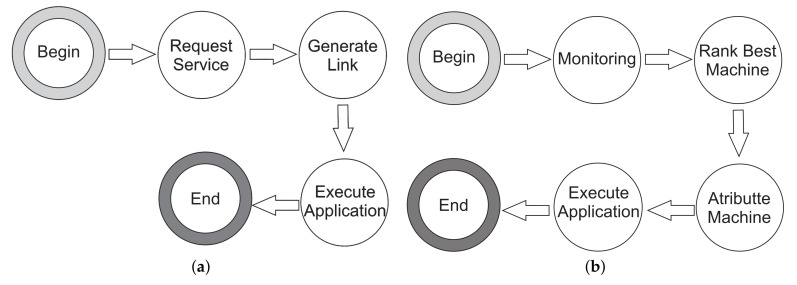
Flow of the system. (**a**) Client interaction flow, (**b**) Flow of internal server processes.

MoHRiPA has two flows: one from the client’s point of view and the other in the provider’s internal processes. The first one comprises the client’s service request process, as shown in Figure 3a. It begins with the request for some service. It finishes with the client accessing the service by using the link access generated by MoHRiPA architecture once the provider has the service available.

Figure 3b depicts the second flow. They were considering the hosts monitored by an agent deployed in the infrastructure and the performance metrics stored on a relational database for which its elastic resource manager performs the auditing and mapping of the performance metrics of this database by using the proposed algorithm called HashRefresh. This algorithm is a hash table sorter updated by request to select the best host with the lowest CPU or memory usage. Nevertheless, the provider assigns service from the client, and this flow ends when the client has services allocated.

**Figure 4:**
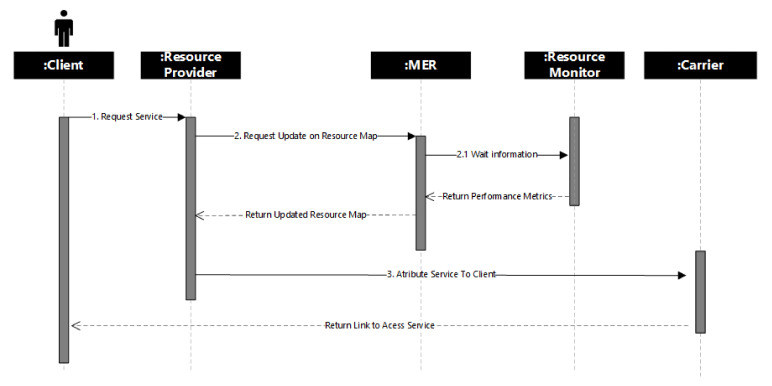
Sequence diagram of architecture.

Figure 4 describes the communication and interaction of both Client Interaction and Internal Server Process flows. It demonstrates the actions of each independent module. The implemented solution performs an accurate performance analysis under the operations of the architecture in order to collect metrics of the infrastructure (RM), analyze resource mapping (MER), and then to provide services to the client (RP).

### 3.1. Resource Provider (RP)

The RP’s role is to receive requests and respond to them based on the MER schedule. This entity has some assistants responsible for allocating the resources (virtual machines) and provisioning the services. The allocation component works in a hybrid manner independent of the virtualization tool, which allows the execution of basic commands such as switching on, switching off, pausing, and returning. The service provider as a carrier component is responsible for providing the link of any service requested by the user or assigning a virtual machine.

### 3.2. Manager of Elastic Resources (MER)

The MER component controls how auditing resources and scales according to the performance metrics collected by the resource monitor functions. CPU-load generates this performance metric imposed in infrastructure, and after MER generates the list of least utilized resources, it forwards it to the Broker, which dispatches the requests according to the availability of resources of the MER list.

MER generates a rank comprising the least utilized resource and forwards it to the Broker, dispatching the requests accordingly. The algorithm for sorting the available resources is up to the user’s choice and serves as a scheduling list in the Broker. Therefore, the execution time requires an algorithm to operate. For this purpose, we proposed tHashRefresh, which is responsible for classifying the physical and virtual hosts and runs on MER.

The HashRefresh is an ad hoc sorting algorithm developed to map the infrastructure resources refreshed by request in terms of complexity, equals an O(log n), depending on the size of the hash table. This algorithm performs a query on a database (line 2 in Algorithm 1) for which its architectural performance information (Performance Index; host) is stored by ordering the best resources and saving it in the hash table. However, the table update occurs for every request that arrives in the architecture, thus maintaining an updated database (lines 3–5). Therefore, there is no waiting on the Broker’s part for a mapping. Furthermore, after hashSort (line 6), the algorithm always keeps the resource pool; therefore, there is no need to wait.
**Algorithm 1** HashRefresh **Input:** Performance Metrics **Output:** Sorted HashMap1:**for all**Request**do**2: 
data←SQLQuerry3: **while** data.next() **do**4:  
hash←host,PerformanceIndex5: **end while**6: 
hash.Sort(value.PerformanceIndex)7:**end for**

In addition to the HashRefresh, MER may also run other algorithms such as ListScheduling, which is an algorithm used to rank resources in heterogeneous environments, thus assessing a consistent resource pool even with an infrastructure with different capacities [28]. ListScheduling updates the resource pool in terms of periodic time or when the list is empty (line one) in our solution, and then this resource list is valid only for 10 s. The Algorithm 2 performs a SQL search for performance information (Performance Index; host); then, when a resource mapping is performed (line 2), the list can be consumed in those 10 s. After that, the list is sorted and updated (line 3), and we can analyze the cadence of the list of resources.
**Algorithm 2** ListScheduling **Input:** Performance Metrics  **Output:** Sorted List1:**while**list.isnotEmpty**OR**cadencelistTime<= 10 s **do**2: 
list←SQLQuerry(host,PerformanceIndex)3: 
list.Sort(value.PerformanceIndex)4:**end while**

### 3.3. Log Server (LS)

The LS component records the system log. The log server in a cloud infrastructure is crucial, as all the information is needed to identify possible problems and is needed to store the results of interactions between the components. The information processing time, the number of requests processed, and providers that answered those requests are saved into a relational database. This log is responsible for analyzing the performance evaluation section data, as will be discussed in the following sections of this paper.

### 3.4. Resource Monitor (RM)

The RM component is responsible for monitoring resources such as CPU, memory, disk, and network. In addition, this module collects performance metrics used by the resource auditor (MER) in order to calculate the metrics of the hosts available in the private cloud and define which will be the best host within the provider in order to meet the client’s request.

An essential point in this work is related to the performance metrics used to classify computational resources. Metrics such as CPU and Memory are generally used in most classification and ranking systems as a specific set of data and do not consider the total performance of a physical machine.

(1) $$ID=\sqrt{I_{CPU}^{2}+I_{MEM}^{2}+I_{NET}^{2}+I_{DISK}^{2}}$$

In this manner, we used the metric represented by Equation (1) of [27] for the correct mapping of computational resources in the index. The proposed performance metric generates a performance index in both homogeneous and heterogeneous resource environments. The Equation (1) performs a normalization of N performance metrics (in our case, CPU, MEM, NET, and DISK). These metrics measure their Euclidean distance from the idle state (all indicators with value zeroes); therefore, we can obtain a weighted performance index vector (PIV) by squaring all the terms to describe the resource’s overall utilization rate. As more values near one in the PIV, the more busy the resource is; if there are more zeroes, then the resource is idler. After this normalization, we obtain a number representing the composite metric and prioritize it, obtaining more accurate information regarding the computational resources consumption of the physical host. In this manner, the assumption is to perform precise scheduling to improve the architectural resources.

## 4. Performance Assessment

The performance evaluation aims to analyze the behavior of the workload in any architecture; for this end, our evaluation method followed the foundation of [29]. Therefore, the experimental design, factors, and levels were defined and studied very carefully, exploring the main points of analysis, such as the correlation of the mapping algorithms and the communication of the architecture modules. We have to use the CPU-Bound application in this experiment, which is fundamental for composing the metrics necessary for generating the workload. This load emulates the CPU-load in the architecture and generates the ranking.

The main metrics to be analyzed are average response time, throughput, individual request time, and individual time for all independent modules. When obtaining these times, it is possible to analyze the data and measure where architectural bottlenecks occur, the behaviors of the different experiments, and how they are related, among others.

Thus, the design of experiments can obtain accurate information about the whole system which consists in open discussion about what causes low-performance and what influences most systems [29]. As it is a service-oriented architecture, the impact of requests can impair service at the provider [30]. As a result, it is essential to set up a suitable testing environment and model the primary factors and levels to be analyzed by using the complete factorial model.

The factors are defined to analyze service attendance so that the workload will test the performance of algorithms. A time of three seconds between requests time was assigned as an analysis parameter to determine how the system behaves with less time. In the case of 5 s between requests, the interval is more significant, generating minor system overhead, which allows measuring how much the arrival time between requests impacts the architecture. Table 2 shows the fixed parameters used to configure Jmeter for all experiments.

The margin of confidence of experiments was performed ten times, with 100 requests for each execution of eight experiments. The confidence interval is applied and defined by Minitab adjusting the Bonferroni correction to maintain the simultaneous confidence level at 95%. This value corresponds to the limit of homogeneous virtual machines with a configuration in which each VM has one VCPU and 4Gb RAM. This configuration is based on Amazon (https://aws.amazon.com/en/ec2/instance-types/ acessed in 19 August 2021) instances and follows as an example of m6g.medium and Google Cloud instances (https://cloud.google.com/compute/docs/instances acessed in 19 August 2021) that correspond to n1-standard-1.

### 4.1. Algorithms Used

Two statistical distributions were selected to assess the arrival behavior of requests: a uniform distribution due to regularity and the other exponential distribution due to exponential behavior. Another factor is the time between requests, which determines the behavior of the distributions. Finally, two mapping algorithms were adopted, namely ListScheduling and the proposed HashRefresh (Table 3).

This design produces a result of the complete factorial, so we performed eight experiments ($2^{3}$—3 factors with two levels each) that were defined to accurately analyze the architecture results, as shown in Table 4. MoHRiPA has two aspects of service, service assignment and allocation of virtual machines. In this analysis, we executed the service attendance scenario. Among the architecture’s services, we have to use the CPU-Bound application in this experiment, which is fundamental for composing the metrics necessary for generating the workload. This load emulated the CPU load in the architecture and generated the ranking [31].

We defined the adoption of the 3 and 5 s on time request during the pre-test phase. We carried it out before the execution of the experiments. This warm up helped determining the time intervals that the architecture would have a higher or lower load. Based on this reflection, we defined the heavy load as 3 s and the light load corresponds to 5 s.

### 4.2. Environment Configuration

The experiments were conducted in the Laboratory of Distributed Systems and Concurrent Programming at the University of São Paulo, Brazil (http://infra.lasdpc.icmc.usp.br/ acessed in 19 August 2021), which was equipped with the clusters Andromeda and Halley. Table 5 shows the cluster’s description.

The infrastructure host has a total of 26 independent nodes. Four of them are reserved for MER, Broker, Monitor, and logServer. The other 22 nodes hosted VMs. A total of eight virtual machines per node were used and distributed between clusters Andrômeda and Halley. There is a module in each VMS called *ClusterLoader* that stores different types of applications. Each application focused on a specific type of processing (CPU-bound, Memory-bound, or Hybrid-bound).

The execution of these applications is fundamental to the functioning of the prototype. These executions influence the provider’s monitoring, a variation in the consumption of resources necessary for ranking the MER. A monitoring tool called *Dstat* (http://dag.wiee.rs/home-made/dstat/ acessed in 19 August 2021) was also used, which queries about the performance metrics in /proc. By using another tool developed for the work (*MonitorAPi*), it performs the treatment of data from *Dstat*. *MonitorAPi* aims at performing the treatment in any monitoring tool when configuring the specific string treatment for each tool.

The workload generation comprises statistical distribution, assignment of requests, and time between requests performed by *Apache Jmeter* (https://jmeter.apache.org/ acessed in 19 August 2021). This tool generated the workload and made supplementary resources available as a monitor of individual requests, which made it possible to obtain information related to the flow and response time of the providers. We used *jmeter* to request a service from an existing VM in infrastructure.

## 5. Result Analysis

**Figure 5:**
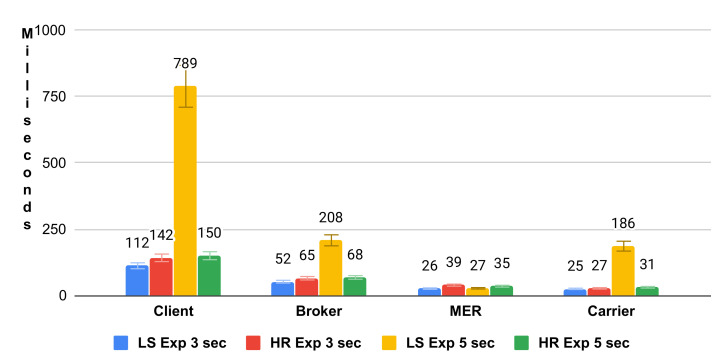
Average uniform exponential time.

Figure 5 shows a bar graph referring to the exponential distribution. The HashRefresh (HR) and ListSheduling (LS) Exp 5 s presented longer average time in all modules. These approaches have constant request updates, corroborating with the increase in average time.

**Figure 6:**
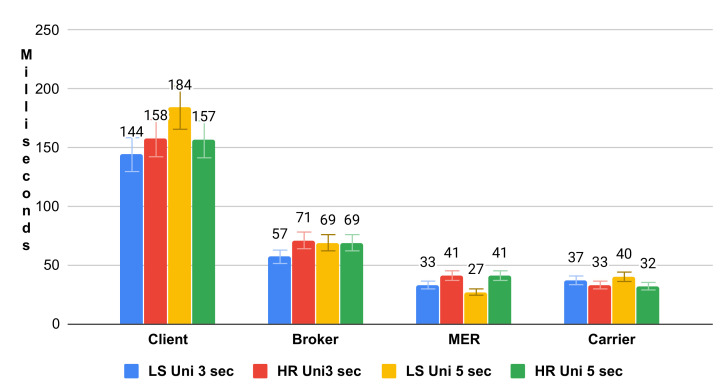
Average Uniform Response Time.

**Figure 7:**
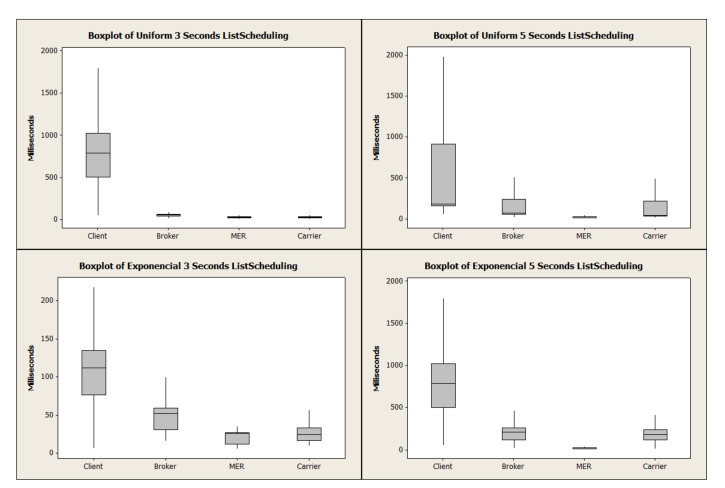
Average of ListScheduling time distribution.

**Figure 8:**
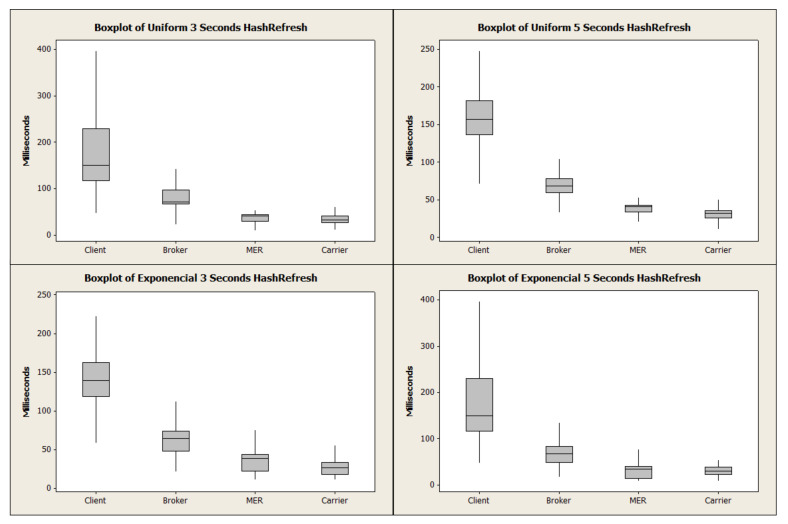
Average of HashRefresh time distribution.

Figure 6 depicts the uniform distribution bar graph. Due to the behavior of this load concerning the average time, it is observed that HashRefresh spent a longer period of time in broker and MER and is close to ListScheduling in the Client (request-response) and Carrier due to the number of requests. ListScheduling demonstrates that the exponential distribution overloads the broker. ListScheduling demonstrated a superior response time in Client and Carrier due to the load distribution in the uniform distribution. The algorithm adapts the requests depending on the periodic list update time. To extend the analysis, Figure 7 and Figure 8 represent the boxplot of the experiments carried out.

**Figure 9:**
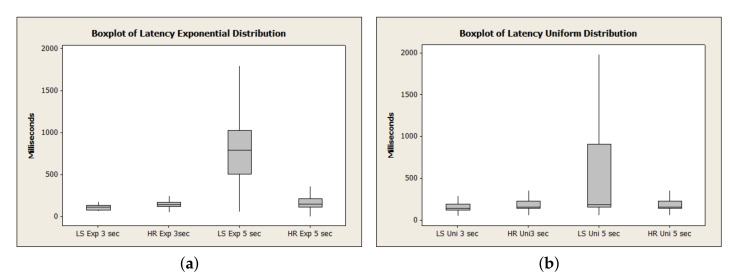
Total latency. (**a**) Latency time distribution: exponential, (**b**) Latency time distribution: uniform.

Figure 9a,b show the total latency. We highlight that, as the time between requests increases, the module MER heavily influences the client since all the mapping centralized in this module generates a bottleneck in the customer’s response time. In ListScheduling, the update rate, when the time between requests is 5 s, corresponds to a high latency; this behavior is due to how the algorithm works, which concludes that the list’s rate can be very high. Table 6 demonstrates the dispersion of the mean and the variance concerned and the required latency to meet an allocation request in which we can observe that the ListScheduling methods have a higher mean. However, HashRefresh has a greater variance due to the frequency of updating the map.

Eventually, requests can arrive during this period of the list, thus increasing the time to fulfill the request. Meanwhile, compared with the HashRefresh algorithm, is does not have high latency. After all, this algorithm does update by requests to keep the mapping continuously updated, thus increasing the service capacity for not having this periodical mechanism.

Regarding the attribution of services, after due analysis, the most significant bottleneck point of the architecture was identified, which was MER, and it responsible for the mapping of resources. MER is very requested and generates much latency due to its response to the *broker*, which may represent an increase in service time. With respect to resource mapping algorithms, the LS’s performance was 7% lower in the high-cadence uniform list scenario than the HR due to its update difference. While LS has a list with time to update the map, HR updates its hash table at each request, overloading broker modules (Carrier; Allocator) in 24% in the uniform scenario and 25.5% in the exponential scenario due to its high demand. Regarding overall performance, HR has a higher performance in responding to the broker and generates much overhead than compared to LS. When we analyzed the bar graph, HR increased compared to LS when the time variation is less because LS updates less during HR. To finalize the HR, a 50% increase in total MER/Broker latency due to the refresh method was observed.

## 6. Conclusions

The need for intelligent resource management is increasingly evident due to the massive adoption of infrastructure automated provisioning. The bottleneck identification plays a crucial role in improving existing cloud computing architectures, enabling services to reach new scalability levels. Our work demonstrated how the workload impacted the computing architecture; individual modules present a viable approach to isolating computing functions and mapping them to resources to achieve scalability. Our solution assumes a resource provider to allocate computing units and a manager to control elasticity. We demonstrated that the MER module is responsible for mapping the resource pool and influences the total response time. Therefore, this module can also be considered the biggest bottleneck in the architecture. This work proposes the HashRefresh mechanism for mapping resources.

Two resource mapping algorithms were analyzed, namely ListScheduling and HashRefresh. The ListScheduling algorithm performance was inferior to HashRefresh due to their differences in approach. While ListScheduling has a list with time to update the map, HashRefresh performs updates to its hash table with each request. Thus, HashRefresh presents better results regarding overall performance, but it also generates more overhead than the ListScheduling algorithm. Despite the subtleties of the resource mapping algorithms, the periodic time of the ListSchdeuling list is a factor that significantly influences response time. However, it is worth mentioning that HashRefresh had a shorter period of time due to its constant updating structure. Our approach presents an exploratory study on scalability issues on cloud computing architectures, aspects of related work, and the modularization of architecture. We also employed formal performance evaluation techniques and designed a complete factorial experiment to identify the influences of levels and factors that were essential for assessing the behavior of architecture concerning specific configurations.

As future work, we aim to analyze a different number of customers and distributed brokers. Our analysis considered only one JMeter client in this work, which impacts the broker from the same source of requests. Regarding the analysis of the impact of factors, we concluded that the customer was severely affected. A new configuration of experiments to distribute the JMeter on more machines can help create better services. As a complement, we aim to analyze the impact of implementing a load balancing module that replicates a set of requests for more than one broker.

## Figures and Tables

**Table 1:** Resource management approaches.

Author	Approaches				
	Resource Management	Resource Monitor	Economic Policies	Scheduler	Environment
[15]	Service	no	no	FIFO, Round-Robin	Simulator
[16]	Allocation	no	no	Deep Learning	Simulator
[17]	Allocation	no	no	Queueing Theory	Simulator
[19]	Allocation	no	no	Analytic hierarchy system	Simulator
[20]	Allocation	no	yes	Differential Evolutionary Algorithm (DEA)	Simulator
[21]	Migration	yes	no	Markov chain	Simulator
[23]	Allocation	yes	yes	Deep Reinforcement Learning	Simulator
[24]	Allocation/Migration	yes	yes	List Scheduling	Simulator
[25]	Allocation/Migration	yes	yes	Leader Election	Simulator
MoHRiPA	Allocation	yes	yes	HashRefresh (proposed)	Prototype

**Table 2:** Fixed parameters that define all experiments.

Fixed Parameters	
Number of Clients	1
Ramp-Up Period	1 s
Number of Iterations Per Thread	100
Number of Replication	10

**Table 3:** Table of factors and levels.

Factor	Level
Statistical Distribution	Uniform; Exponential
Time between Requests	3 s, 5 s
Mapping Algorithm	Hashrefresh; ListScheduling

**Table 4:** Table of Experiments.

Experiment	Distribution	Time Between Requests	Algorithm
A	Uniform	3 s	HashRefresh
B	Uniform	5 s	HashRefresh
C	Exponential	3 s	HashRefresh
D	Exponential	5 s	HashRefresh
E	Uniform	3 s	ListScheduling
F	Uniform	5 s	ListScheduling
G	Exponential	3 s	ListScheduling
H	Exponential	5 s	ListScheduling

**Table 5:** Configurations of infrastructure.

Clusters		
	**Andrômeda**	**Halley**
Processor	AMD Vishera 4.2 Ghz	Intel Core I7 3.60 Ghz
Storage	32 GB RAM, SSD 480 GB and HDD 2 TB	32 GB RAM, SSD 480 GB and HDD 2 TB
Nodes	13	13

**Table 6:** Experiment latency statistical metrics.

Experiment	Mean	Variation	Standard Deviation
LS Exp 3 s	110	93.3	11.8
HR Exp 3 s	140	305.5	117.0
LS Exp 5 s	789.5	505.7	200.6
HR Exp 5 s	150	515.9	258.7
LS Uni 3 s	144	371	160.5
HR Uni 3 s	155.5	275.1	84.5
LS Uni 5 s	184	528.7	243.7
HR Uni 5 s	153	440.8	203.5

## Data Availability

Data sharing can be found on https://github.com/Tomiatti/Mohripa-msc/, accessed on 19 August 2021.
